# Performance of multiple neural networks in predicting lower limb joint moments using wearable sensors

**DOI:** 10.3389/fbioe.2023.1215770

**Published:** 2023-07-31

**Authors:** Zainab Altai, Issam Boukhennoufa, Xiaojun Zhai, Andrew Phillips, Jason Moran, Bernard X. W. Liew

**Affiliations:** ^1^ School of Sport, Rehabilitation and Exercise Sciences, University of Essex, Essex, United Kingdom; ^2^ School of Computer Science and Electronic Engineering, University of Essex, Essex, United Kingdom; ^3^ Department of Civil and Environmental Engineering, Imperial College London, London, United Kingdom

**Keywords:** machine learning, wearable sensors, joint moments, motion capture, musculoskeletal modelling

## Abstract

Joint moment measurements represent an objective biomechemical parameter in joint health assessment. Inverse dynamics based on 3D motion capture data is the current 'gold standard’ to estimate joint moments. Recently, machine learning combined with data measured by wearable technologies such electromyography (EMG), inertial measurement units (IMU), and electrogoniometers (GON) has been used to enable fast, easy, and low-cost measurements of joint moments. This study investigates the ability of various deep neural networks to predict lower limb joint moments merely from IMU sensors. The performance of five different deep neural networks (InceptionTimePlus, eXplainable convolutional neural network (XCM), XCMplus, Recurrent neural network (RNNplus), and Time Series Transformer (TSTPlus)) were tested to predict hip, knee, ankle, and subtalar moments using acceleration and gyroscope measurements of four IMU sensors at the trunk, thigh, shank, and foot. Multiple locomotion modes were considered including level-ground walking, treadmill walking, stair ascent, stair descent, ramp ascent, and ramp descent. We show that XCM can accurately predict lower limb joint moments using data of only four IMUs with RMSE of 0.046 ± 0.013 Nm/kg compared to 0.064 ± 0.003 Nm/kg on average for the other architectures. We found that hip, knee, and ankle joint moments predictions had a comparable RMSE with an average of 0.069 Nm/kg, while subtalar joint moments had the lowest RMSE of 0.033 Nm/kg. The real-time feedback that can be derived from the proposed method can be highly valuable for sports scientists and physiotherapists to gain insights into biomechanics, technique, and form to develop personalized training and rehabilitation programs.

## 1 Introduction

Joint moments are an indirect measure of internal joint forces (Stensgaard Stoltze et al., 2018; Holder et al., 2020) and have multiple clinical applications such as injury risk assessment (Kiesel et al., 2007) and rehabilitation (Liu et al., 2018). In biomechanics, inverse dynamic (ID) is the current gold standard approach to calculate joint moments (Koopman et al., 1995; Forner-Cordero et al., 2006; Winter 2009) using data collected by an optical three-dimensional (3D) motion tracking system with force plate measurements. Yet, the use of such methods is limited to a laboratory environment (Simon, 2004), which makes it challenging to be applied in a clinical or field setting.

To overcome the limitation of traditional methods, recently, machine learning approaches have been increasingly used to quantify joint moments based on kinematic variables (Xiang et al., 2022). Studies have used several sources to obtain these variables (Table 1) ranging from: marker-based optical cameras of 3D measurements (Johnson et al., 2019a; Boswell et al., 2021; Liew et al., 2021; Boukhennoufa et al., 2022) or 2D measurements (Dingenen et al., 2015; Boswell et al., 2021), wearable sensors such as electromyography (EMGs) (Xiong et al., 2019; Camargo et al., 2022), inertial measurement units (IMUs) (Stetter et al., 2020; Wang et al., 2020; Camargo et al., 2022), and electrogoniometers (GON) (Camargo et al., 2022) (Table 1). Optical motion capture systems are limited largely to a laboratory setting and are expensive, time-consuming to use, and subjected to errors associated with marker placement. In contrast, a machine learning approach based on wearable sensors allows for possible measurements outside laboratory settings. Camargo et al. used combined EMG and IMU sensor data with machine learning to estimate hip, knee, and ankle joint moments (Camargo et al., 2022). Still, EMG sensors are very sensitive to sensor placement (e.g., crosstalk problems) and so require high expertise, in addition to time delay estimation issues. On the contrary, IMU is a cost-effective, relatively portable method of measurement that is not limited to a laboratory setting and can be accessed in resource-constrained environments. Importantly, machine learning based on IMU sensor data alone has shown good accuracy when predicting knee joint moments (Stetter et al., 2020) during walking and running. However, to the author’s knowledge, no study has used IMU sensors alone to estimate joint moments in the lower limb (hip, knee, ankle, and subtalar).

**TABLE 1:** List of literature studies used machine learning to estimate lower limb joint moments.

Study	Locomotion tasks	Predictors	Joint moment	Discrete/Continuous	Machine learning algorithms	Validation method	Performance
Johnson et al. (2019b)	Walking, running, and sidestepping	Marker trajectories	Knee	Peak moment	Convolutional neural network	80% training, 20% testing of each task	*RMSE (Nm/kg)*
							Knee flexion: (0.67 ± 0.24)
							Knee adduction: (0.53 ± 0.25)
Liew et al. (2021)	Running	3D angle, velocity, and acceleration of the joint	Hip, knee, ankle, and subtalar	Waveform	Functional regression, deep neural network^*^, and transfer learning	80% training, 20% testing overall dataset	*RMSE (Nm/kg)*
							Hip flexion: (0.24)
							Hip adduction: (0.19)
							Knee flexion: (0.25)
							Ankle plantarflexion: (0.16)
							Subtalar inversion: (0.07)
Stetter et al. (2020)	Walking, running, cutting manoeuvre	IMU signals	Knee	Peak moment	Fully connected neural network with 2 hidden layers	leave-one-subject-out cross-validation	*RMSE (Nm/kg)*
							Knee flexion: (0.58–1.13)
							Knee adduction: (0.37–0.8)
Wang et al. (2020)	Walking	IMU signals	Knee	Waveform	Fully connected neural network with 2 hidden layers^*^, and XGBoost	80% training, 10% testing, and 10% validation of subjects	*MAE%*
							Knee adduction: 0.002
Xiong et al. (2019)	Treadmill walking	EMG signals	Hip, knee, and ankle	Waveform	Neural network with 3hidden layers	Split based on trials overall subjects	*NRMSE%*
							Hip flexion: (7.8944)
							Hip adduction: (6.235)
							Knee flexion: (6.235)
							Ankle plantarflexion: (6.6967)
Boswell et al. (2021)	Walking	Marker trajectories	Knee	Peak moment	Fully connected neural network with 10 hidden layers	80% training, 10%− development, and 10% testing sets of subjects	*MAE %*
							Knee adduction 0.53
Camargo et al. (2022)	Treadmill walking, ascent/descent of stairs and ramps	EMG and IMU signals	Hip, knee, and ankle	Waveform	Fully connected neural network with 2 hidden layers, and XGBoost^*^	cross-validation based on trails per subject	*MAE (average of all joints Nm/kg)*
							0.06 ± 0.02
Boukhen-noufa et al. (2022)	Walking	3D angle, velocity, and acceleration of segment centre of mass	Knee	Impulse	Deep neural network (Baseline model, InceptionTime^*^, Transfer learning, TS-ResNet, GADF-xResnet)	75% training, 25% testing, and 10% validation overall dataset	*RMSE (Nm.s/kg)*
							2.46

^*^ Reported performance, RMSE: root mean squared error, MAE: mean average error, NRMSE: normalized root mean squared error.

The majority of previous machine-learning studies have focused on estimating joint moments during walking (Xiong et al., 2019; Boswell et al., 2021; Boukhennoufa et al., 2022; Camargo et al., 2022) or running (Liew et al., 2021), whilst some have also considered cutting maneuvers (Johnson et al., 2019b; Stetter et al., 2020) or turning (Stetter et al., 2020). However, dynamic movements such as this do not necessarily reflect the type of ambulation that the general population engages in while executing conventional daily tasks such as climbing stairs and descending ramps. While climbing stairs can often be regarded as a facile activity for young healthy individuals, it can be a challenging task for elderly individuals. This represents a clear gap in the literature as previously highlighted by Camargo et al. (2022) who have recommended the extension of applications beyond walking and running.

The type of machine learning algorithms used to predict joint moments influences its prediction performance. Neural networks are the most common algorithm used for joint moment prediction (Liew et al., 2021; Xiong et al., 2019). Different neural network approaches have been used and range from a shallow network with one or two hidden layers (Stetter et al., 2020) and boosting (Wang et al., 2020; Camargo et al., 2022) to a deep neural network (Boswell et al., 2021; Boukhennoufa et al., 2022; Liew et al., 2021; Wang et al., 2020). Shallow networks such as Ensemble learning, and Support Vector Machines (SVM) have certain drawbacks. First, they work better with relatively small data sets. Second, those approaches are very sensitive to imbalanced data. Furthermore, shallow neural networks work well for predicting scaler values and cannot accommodate temporal variables, therefore researchers have opted to treat each value of a time-series as an independent observation (Stetter et al., 2020). While deep neural network structures introduce advantages in processing time-series sensor data and require lower computational costs than traditional machine-learning approaches (Xiang et al., 2022). A few new deep neural networks were proposed as advanced time series architectures for prediction and/or classification and regression problems. For example, InceptionTime architecture predicted knee abduction impulse during walking with 8.28% absolute mean square error (Boukhennoufa et al., 2022). Another new proposed architecture is eXplainable convolutional neural network (XCM) which outperforms the state-of-the-art of Multivariate Time series classifiers such as Long Short-Term Memory (LSTM) (Fauvel et al., 2021) when evaluating its classification performance on public UEA datasets (Bagnall et al., 2018). While Time Series Transformer (TST) reported with either the best or the second best classification performance compare to other models such as XBoost, Inception, and ResNet when testing on several public time series data sets from various domains (Zerveas et al., 2021). Recurrent neural network (RNN) is commonly used in temporal problems (e.g., speech recognition) as it takes information from prior inputs to influence the current input and output. Considering the recorded superior classification performance of these architectures in real-world data, yet not in biomechanics, the current study explores the ability to use these new methods in estimating lower limb joint moments based on IMU sensor data.

The current study aims to evaluate the performance of recently proposed deep neural network architectures on estimating lower limb joint moments during different locomotion modes using inputs from inertial wearable sensors only. Since the success of a model is measured by its ability to generalize to new data (Halilaj et al., 2018), it is therefore critical for a machine learning model performance to be assessed on held-out data (observations) not used in the training (Xiang et al., 2022). Therefore, to assess the effect of model training with and without held-out observations at a subject level, the investigated architectures were tested on two different methods. First the “Typical-split” method: where the recorded data was randomly split into training and test sets, allowing data from each subject to contribute to both the training and test sets; and second the “Leave-subjects-out” method: where all the records of each subject are randomly assigned as a group to either the training set or to the test set. The real-time feedback that can be derived from the proposed method can be highly valuable for sports scientists and physiotherapists to gain insights into biomechanics, technique, and form to develop personalized training and rehabilitation programs.

## 2 Material and methods

### 2.1 Dataset

**FIGURE 1:**
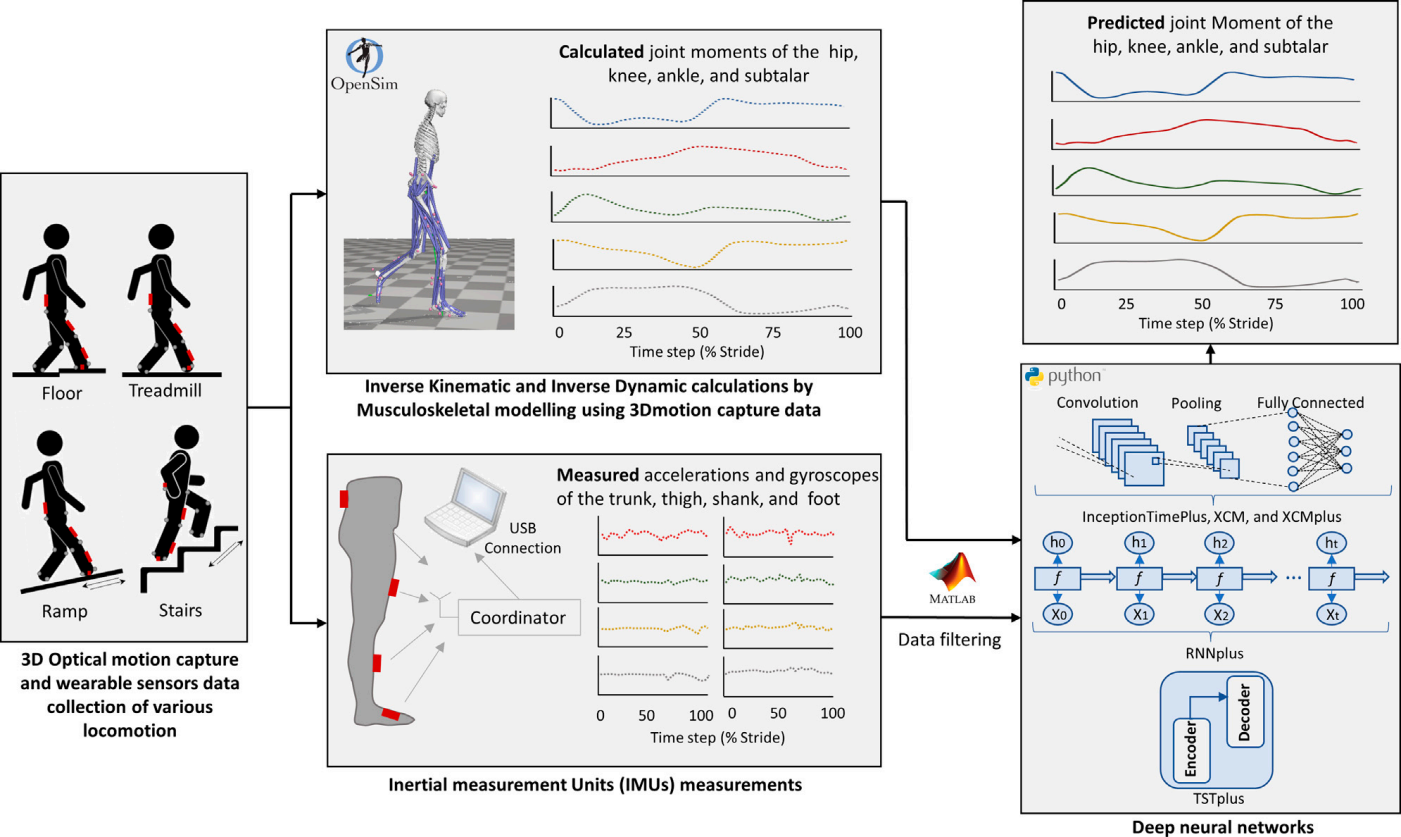
Illustration of the Study workflow. Data collection included 3D motion capture data (marker trajectories and ground reaction forces) and four Inertial measurement units (IMUs) (accelerations and gyroscopes) at the trunk, thigh, shank, and foot (Camargo et al., 2021). Joint moments of the hip, knee, ankle, and subtalar was calculated by musculoskeletal models in OpenSim. Strides with outlier data were excluded from the study. Five different deep neural networks (InceptionTimePlus, XCM, XCMplus, RNNplus, and Time Series Transformer plus (TSTPlus)) were trained to predict joint moments (outputs) using the measured accelerations and gyroscopes (inputs).

The entire workflow of the current study (starting from data collection up to joint moments prediction by machine learning models) is illustrated in Figure 1. The dataset used in this study is from an open-source biomechanics database (Camargo et al., 2021). The study involved 22 healthy adults with average age of 21 ± 3.4 years, body mass 68.3 ± 10.83 kg, and height 1.70 ± 0.07 m, full details of each participant can be found in the original open-source publication (Camargo et al., 2021).

Motion capture (200 Hz, Vicon. Ltd., Oxford, Uk), Ground reaction forces (GRF) (1,000 Hz, Bertec, Ohio, United States), and inertial measurement units (IMUs) (200 Hz, Yost, Ohio, United States) data of all the 22 participants were used in this study. A set of 32 motion markers were used for the lower body part, while four IMUs were placed on the trunk, thigh, shank, and foot of the right leg. Data of each participant were collected for multiple locomotion modes: level-ground walking, treadmill walking, stair ascent, stair descent, ramp ascent, and ramp descent. Level-ground walking was captured at three different self-selected speeds with five clockwise and five counterclockwise circuits, while treadmill walking data were recorded for 28 different speeds with a range of 0.5–1.85 m/s and 0.05 m/s increment. For stair ascent and stair descent modes, four different stair heights (102 mm, 127 mm, 152 mm, 178 mm) of a 6-step staircase were used and five sets of trials were executed for each height with a total of 40 trials for each motion mode. Ramp trials were collected for six different inclinations angles (5.2°, 7.8°, 9.2°, 11°, 12.4°, and 18°) along a 5-m long ramp and, again, five sets of trials for each inclination were undertaken with a total of 60 trials for both ramp ascent and ramp descent modes.

### 2.2 Generating kinetic and kinematic data

In addition to the raw data, Camargo and his colleagues provided an open-access repository with musculoskeletal models (MSKMs) generated in OpenSim (Delp et al., 2007) for all participants, along with MATLAB scripts allowing for easy analyses of the inverse kinetics and inverse dynamics data. In the current study, the repository provided by Camargo’s study was used to extract all the recorded trials from the raw data. Then, strides for all locomotion modes were identified and sampled based on the heel-strike and toe-off phases. Strides were then normalised and sampled at 100-time points for a full gait cycle. For this study, only strides that have associated GRF data were extracted and considered. That was so it is possible to generate the associated kinetic data using musculoskeletal models, which require GRF as an input. Five joint moments were calculated by the musculoskeletal models (hip flexion moment, hip adduction moment, knee flexion moment, ankle plantarflexor moment, and subtalar inversion-eversion moment) and considered in this study. These joint moments were later used as the outcomes in the machine learning analysis. The associated IMUs data of all the selected strides, represented by accelerations and gyroscopes of four segments; trunk, thigh, shank, and foot, were then extracted and used as the predictors in the machine learning analyses. Accelerations and gyroscopes data were recorded in the three planes of motion; sagittal, coronal (frontal), and transverse planes (represented by three axes x, y, and z). Therefore, the total number of the predictors in the machine learning analysis was 24 (number of used IMUs multiplied by accelerations and gyroscopes at the three axes).

The total number of the extracted strides of all participants including all locomotion modes was 27,845 strides. Data were then cleaned in MATLAB (R2021a) by removing the outliers from the extracted strides to prepare them for the machine learning analysis. The process of cleaning the data was done in two stages. During the first stage, the flat signals (or in other words the missing signal) were identified using the standard deviation of the derivatives, and the associated stride was excluded. In the second stage, the outliers in the remaining signals were detected based on the Median Absolute Deviations (MAD) (Leys et al., 2013) and replaced by the next non-outlier value. To account for the variation among individuals, the data cleaning process was done at the participant level, then the cleaned data of all participants were combined to form the final data for the machine learning analysis. The final data included 21,787 strides.

### 2.3 Machine learning modeling

All analyses were performed in Python (version 3.9.0), with packages (Numpy v1.20.3, Pandas v1.3.4, Scipy v1.7.1). All ML models were trained using either Keras (version 2.6.0) or Tsai (version 0.3.1) from fastai with Google Collab.

#### 2.3.1 Data pre-processing

All time-series data of both predictors and outcome were segmented between heel strike and toe-off. And strides were sampled to have a 100-time points for a full gait cycle. The total number of observations in the dataset was 21,787 corresponding to 21,787 participants’ strides. The predictor dataset was organized into a 3D array shape 21787×24×101 , where the second dimension was the number of predictors, and the third dimension was the number of time points. The outcome dataset was organized into a 2D array shape 21787×5×101 , where the second dimension was the number of outcomes, and the third dimension was the number of time points.

Following the purpose of the current study, two approaches were used to generate the training and testing data sets.

(1) Typical-split method: data of the strides of all the 22 participants were combined for both the predictor and outcome datasets, then each set was split into training (80%, n = 16,340) and testing (20%, n = 5,447).
(2) Leave-subjects-out method: split was done at a participant level, where each of the predictor and outcome datasets was split into training set involving strides of 17 participants (n = 17,053) and testing sets involving strides of the left five participants (n = 4,734). The decision of the selected number of participants for training and testing sets was done so that the resulting size of each set is close to the size of training and testing sets using the Typical-split method.

#### 2.3.2 Algorithms

The baseline 2D neural network model architecture can be found in Table 2. The model hyperparameters were selected based on initial exploratory analysis. Neural network weights were initialized with Xavier initialization. The Xavier initialization technique involves generating a random number from a uniform probability distribution (U) that falls between the range of -1n√ and 1n√, where “n” denotes the number of inputs that the node receives $-1/\sqrt{n}$ and $1/\sqrt{n}$ . A batch size of 2048, a learning rate of 3e^−3^, and an Adam optimiser were used. A sensitivity analysis for the selected number of epochs was conducted using three tested epochs (10, 30, and 50). The relative difference of the root mean squared error of the predictions using 10 epochs and 50 epochs was about 75% difference. No considerable reduction of the model error occurred when using more than 50 epochs, and hence, 50 epochs was selected. The mean squared error was used as the loss criteria.

**TABLE 2:** Baseline model architecture and parameters.

Layer	Description	Output size
Input		Batch × 101 × 24
Conv1	Conv2D (N = 64, F = 3 × 3, S = 1, P = valid, A = ReLu)	Batch × 99 × 22 × 64
Conv2	Conv2D (N = 128, F = 3 × 3, S = 1, P = valid, A = ReLu)	Batch × 97 × 20 × 128
Max pooling 2D	Conv2D (2 × 2)	Batch × 48 × 20 × 128
Flattening operation		Batch × 61,440
Dense Layer 1	512, dropout 0.1, ReLu	Batch × 512
Dense Layer 2	256, dropout 0.1, A = ReLu	Batch × 256
Dense Layer 3	101, dropout 0.1, A = Linear	Batch × 101
Xavier initialization, batch size = 2048		
learning rate: 0.003, Epochs: 50		

N: number of filters, F: filter size, S: stride, P: padding, A: activation function.

In addition to the baseline model, five different model architectures were evaluated: InceptionTimePlus, XCM (Fauvel et al., 2021), XCMplus, RNNplus (Conv1d + Stacked LSTM architecture), and Time Series Transformer plus (TSTPlus) (Zerveas et al., 2021). For all deep learning models, the final layer of this network consists of a linear layer with 100 units, to predict the joint moment at each time-point of the gait cycle. Cyclical learning rate method was used to find the appropriate learning rate. The loss was plotted with respect to an increasing value of the learning rate. The learning rate was chosen to be in the interval that resulted in the lowest loss, which was found to be between 1e^−5^ to 1e^−4^. The learning rate took the value of 1e^−5^ at the first epoch and then gradually increased to reach a final value of 1e^−4^ at the last epoch. For all the tested architectures, 50 epochs were selected to allow for direct comparison with the baseline model.

##### 2.3.2.1 InceptionTimePus

The InceptionTimePus model is a collection of deep Convolutional Neural Network (CNN) models that are inspired by the Inception-v4 architecture used in computer vision (Fawaz et al., 2020). The InceptionTime model comprises two distinct residual blocks, each consisting of three Inception modules, as opposed to the conventional fully convolutional layers. The input to each residual block is connected linearly to the following block’s input, creating a shortcut connection. The model then uses a Global Average Pooling layer to average the output of the multivariate time-series across the entire time dimension. Each of the Inception modules includes a bottleneck of 1D CNN layer with 32 output channels, kernel size of 1, and stride of 1 to decrease parameter dimensionality. This is followed by three 1D CNN layers of 32 output channels, kernel sizes of 39, 19, and 9 respectively, padding of 19, 9, and 4, respectively, and a stride of 1 for all cases.

##### 2.3.2.2 XCM

The XCM method extracts both 2D convolution filters for observed variables and 1D convolution filters for time directly from input data, leading to more accurate features and better prediction performance than the sequential approach (Fauvel et al., 2021). However, fully connected layers used in CNN architecture for classification can lead to overfitting and a high number of trainable parameters. To address this, XCM uses 1D global average pooling to reduce the number of parameters and improve generalization ability. The non-fully padded convolution filters used in other methods can lead to imprecise identification of important regions in input data, so XCM uses fully padded filters for better results. The upper part of the model uses 2D convolution filters to extract features per observed variable and is composed of a 2D convolutional block, batch normalization, and ReLU activation layers. The lower part uses 1D convolution filters to extract information relative to time and captures the interaction between different time series. The output feature maps from these two parts are concatenated to form a feature map, which is passed through a 1D convolution block and global average pooling before performing classification with a softmax layer.

##### 2.3.2.3 XCMplus

A variant of XCM, similar to XCM except that the 2D and 1D convolution blocks are in sequence.

##### 2.3.2.4 RNNplus

The concept of integrating a feature extractor into the RNN network was inspired by the approach devised by the UPSTAGE team which secured a 3rd place finish in Kaggle’s Google Brain - Ventilator Pressure Prediction Competition, it consists of employing a Conv1d + Stacked LSTM architecture.

The Time Series Transformer Plus model is a basic encoder-decoder Transformer utilised for time series prediction. Unlike other Transformer models, it does not have any head on top and instead adds a distribution head for probabilistic forecasting. This implies that the model learns a distribution, from which one can sample instead of directly outputting values. It comprises two blocks: an encoder that accepts a context length of time series values as input (known as past values), and a decoder that predicts a prediction length of time series values into the future (known as future values). During training, pairs of (past values and future values) are provided to the model. Along with the raw values, additional features can also be provided to the model such as past time features and future time features, which serve as “positional encodings” for the Transformer encoder and decoder respectively. Static categorical features and static real features can also be used as categorical and real-valued features that are static over time.

#### 2.3.3 Predictive performance

The predictive performance of all the tested architectures using Typical-split and Leave-subjects-out methods was quantified by comparing the five joint moments in the test set, against their predicted values using RMSE and relative RMSE (relRMSE) expressed as a percentage (%) of the average peak-to-peak amplitude for the outcomes (Ren et al., 2008), and Pearson correlation coefficient (cor) (Johnson et al., 2019a; Johnson et al., 2019b).

$$RMSE=\sqrt{\frac{\int_{0}^{T}{\left[u_{obs}\left(t\right)-u_{pred}\left(t\right)\right]}^{2}dt}{T}}$$

$$relRMSE=\frac{RMSE}{0.5\left[\sum_{i=1}^{2}\left({max}_{0<t<T}\left(u_{i}\left(t\right)\right)-\min_{0<t<T}\left(u_{i}\left(t\right)\right)\right)\right]}\times 100\%$$

where *T* is the stance phase period between initial contact and toe-off, while $u_{obs}\left(t\right)$ is the value at the $t^{th}$ time point of the observed outcome, $u_{pred}\left(t\right)$ is the value at the $t^{th}$ time point of the predicted outcome, and *i* represents either the observed or predicted outcomes.

## 3 Results

### 3.1 Predictors and outcomes

The waveform plots of the accelerations and gyroscopes measured by the IMUs, and all joint moments calculated by the musculoskeletal models of the entire dataset can be found in the Supplementary Material (Supplementary Figure S1; Supplementary Figure S2; Supplementary Figure S3). The observed and predicted standard deviation (SD) waveform for all joint moments using Typical-split and Leave-subjects-out methods can be found in the Supplementary Material (Supplementary Figure S4; Supplementary Figure S5).

### 3.2 Machine learning models performance

The performance of the all the tested machine learning models represented by RMSE, relRMSE, and correlation coefficient to predict hip, knee, ankle, and subtalar joint moments can be found in Table 3. Across all joints, the average ±SD of the RMSE for the baseline model was 0.067 ± 0.022 Nm/kg, InceptionTimePlus 0.066 ± 0.015 Nm/kg, RNNplus 0.064 ± 0.015 Nm/kg, TSTPlus 0.064 ± 0.015 Nm/kg, XCM 0.046 ± 0.013 Nm/kg, XCMplus 0.059 ± 0.016 Nm/kg using Typical-split method. While the average ±SD of the RMSE using Leave-subjects-out methods for the baseline model was 0.131 ± 0.034 Nm/kg, InceptionTimePlus 0.159 ± 0.045 Nm/kg, RNNplus 0.151 ± 0.057 Nm/kg, TSTPlus 0.162 ± 0.063 Nm/kg, XCM 0.137 ± 0.042 Nm/kg, XCMplus 0.151 ± 0.053 Nm/kg.

**TABLE 3:** Prediction performance of all the tested machine learning models using typical-split and Leave-subjects-out methods.

Model type	Joint moment	Typical-split			Leave-subjects-out		
		RMSE (Nm/kg)	relRMSE	cor	RMSE (Nm/kg)	relRMSE	cor
Baseline Model	Ankle	0.076	5%	0.989	0.131	9%	0.981
	Subtalar	0.029	14%	0.924	0.072	32%	0.646
	Knee	0.071	7%	0.968	0.145	16%	0.876
	Hip_flexion	0.081	7%	0.966	0.148	13%	0.938
	Hip_adduction	0.078	9%	0.97	0.158	18%	0.949
Inception	Ankle	0.074	5%	0.989	0.151	9%	0.966
Time	Subtalar	0.041	16%	0.855	0.09	30%	0.489
Plus	Knee	0.076	7%	0.963	0.213	21%	0.778
	Hip_flexion	0.074	6%	0.973	0.162	13%	0.917
	Hip_adduction	0.067	7%	0.978	0.178	18%	0.943
RNNPlus	Ankle	0.073	5%	0.99	0.124	8%	0.977
	Subtalar	0.038	15%	0.875	0.088	30%	0.514
	Knee	0.074	7%	0.967	0.239	23%	0.769
	Hip_flexion	0.071	6%	0.976	0.137	11%	0.941
	Hip_adduction	0.065	7%	0.983	0.166	18%	0.952
TSTPlus	Ankle	0.073	5%	0.991	0.147	9%	0.975
	Subtalar	0.038	15%	0.876	0.085	28%	0.583
	Knee	0.075	7%	0.966	0.255	23%	0.791
	Hip_flexion	0.072	6%	0.976	0.138	11%	0.937
	Hip_adduction	0.064	7%	0.983	0.187	20%	0.943
XCM	Ankle	0.051	3%	0.995	0.124	8%	0.982
	Subtalar	0.024	11%	0.945	0.076	28%	0.629
	Knee	0.051	5%	0.983	0.182	19%	0.828
	Hip_flexion	0.055	5%	0.985	0.133	11%	0.94
	Hip_adduction	0.051	6%	0.987	0.17	19%	0.958
XCMPlus	Ankle	0.065	4%	0.992	0.127	8%	0.979
	Subtalar	0.03	13%	0.917	0.076	29%	0.617
	Knee	0.069	7%	0.97	0.216	20%	0.821
	Hip_flexion	0.069	6%	0.976	0.161	13%	0.924
	Hip_adduction	0.061	7%	0.982	0.175	0.191	0.956

Generally, the best prediction performance was found for the XCM model using both Typical-split and Leave-subject-out methods. While the worst performance was found for the baseline model when using the Typical-split and baseline model and TSTPlus when using Leave-subjects-out methods. On average across all joint moments, XCM improved RMSE, relRMSE, and correlation compared to the model with the worst prediction by 31%, 30%, and 2%, respectively when using Typical-split method, and by 16%, 7%, and 3% respectively when using Leave-subject-out method.

The variation of the joint moments for multiple locomotion modes as predicted by the machine learning models using Leave-subject-out method can be found in the Supplementary Material (Supplementary Figure S6; Supplementary Figure S7; Supplementary Figure S8) represented by RMSE, relRMSE, and correlation coefficient.

### 3.3 Typical-split vs leave-subjects-out methods

**FIGURE 2:**
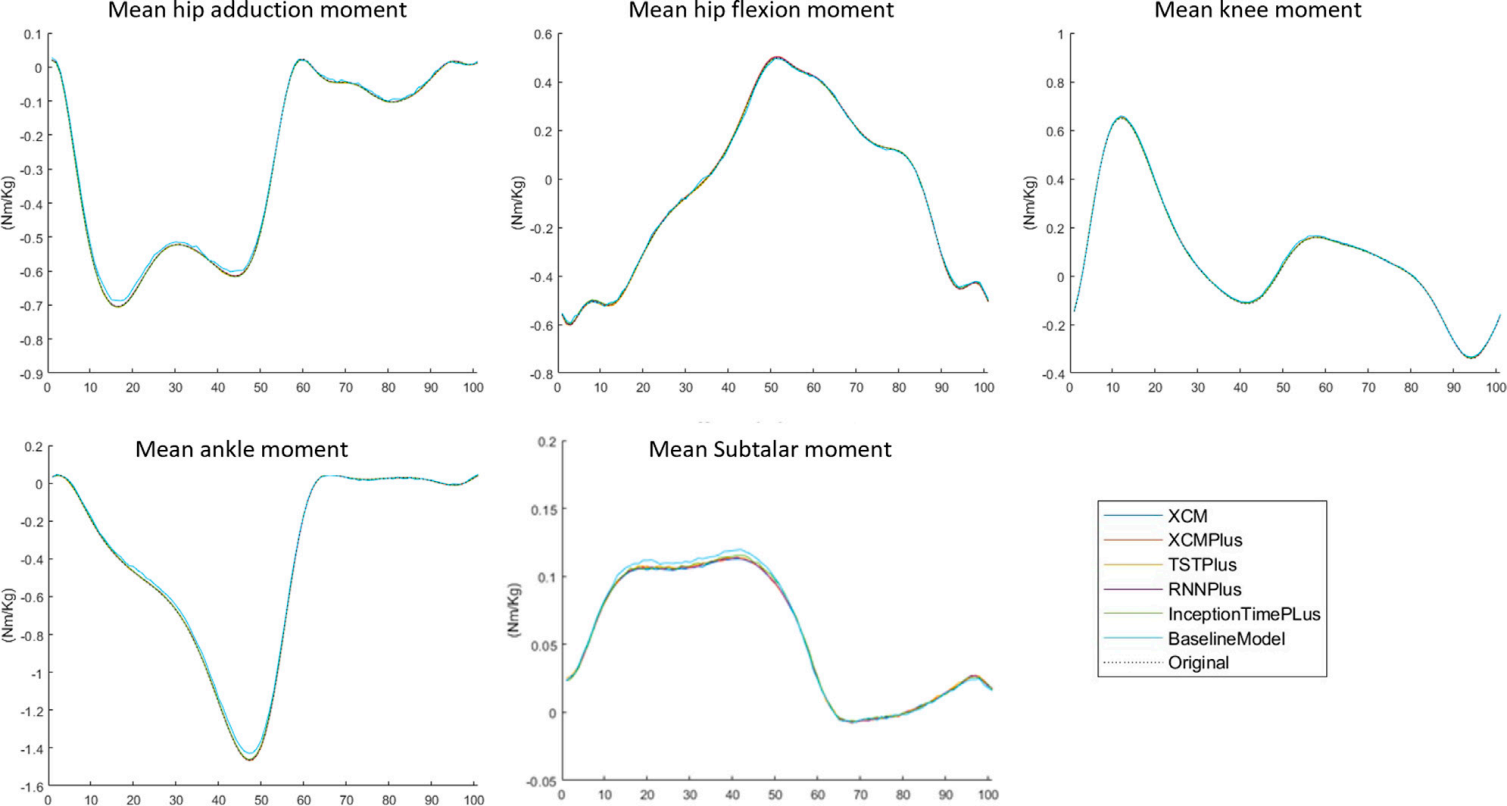
Mean of the predicted joint moment waveforms for the hip, knee, ankle, and subtalar by baseline model, InceptionTimePlus, XCM, XCMplus, RNNplus, and Time Series Transformer plus (TSTPlus) using Typical-split method.

**FIGURE 3:**
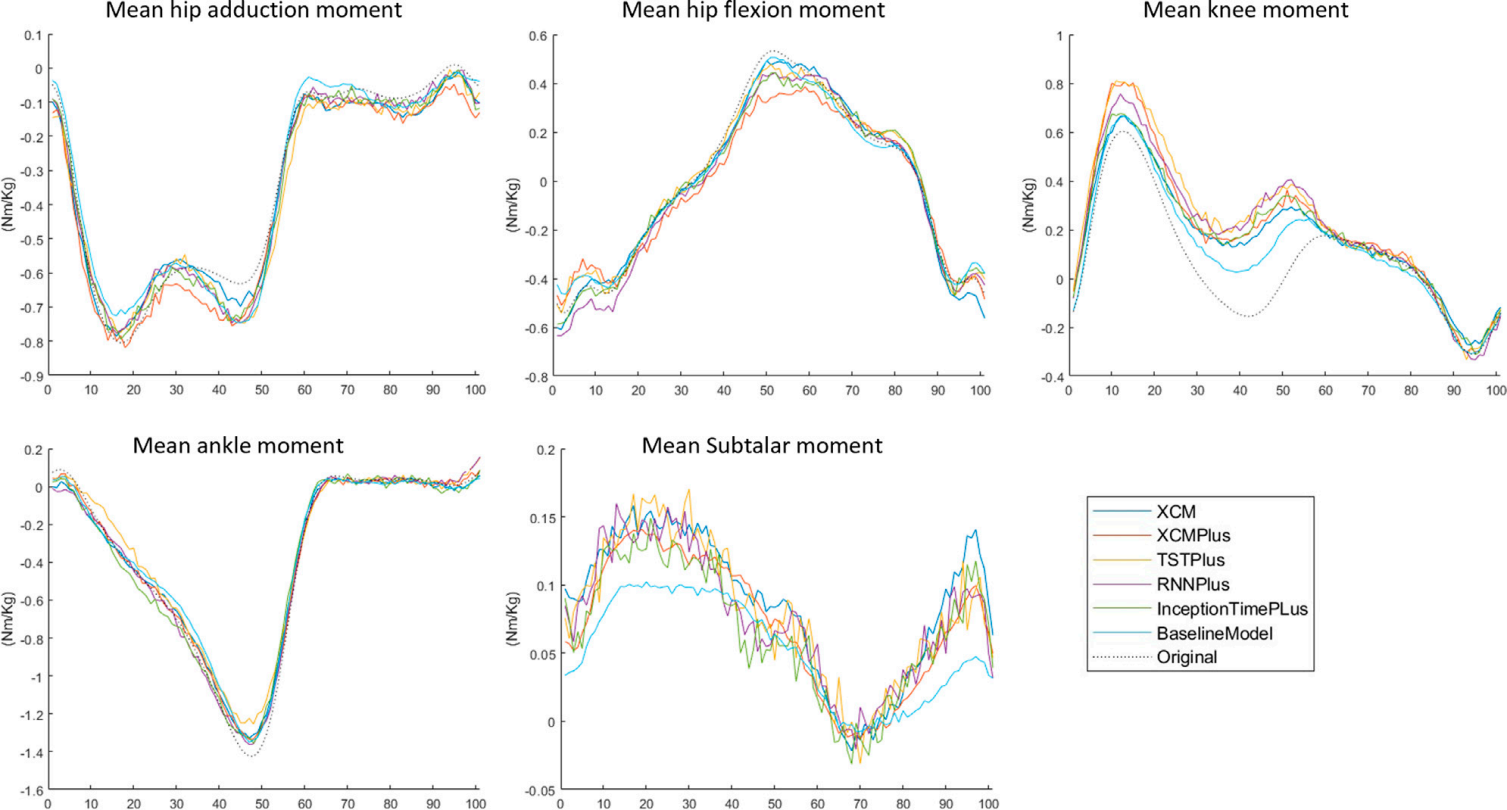
Mean of the predicted joint moment waveforms for the hip, knee, ankle, and subtalar by baseline model, InceptionTimePlus, XCM, XCMplus, RNNplus, and Time Series Transformer plus (TSTPlus) using Leave-subject-out method.

The prediction performance of all the tested machine learning models was reduced when using Leave-subjects-out methods over the Typical-split method (Figure 2; Figure 3). The range (maximum and minimum) of the relRMSE across all the tested machine learning models was 11%–16% for subtalar moment, 3%–5% for ankle moment, 5%–7% for knee moment, 5%–7% for hip flexion–extension, 6%–9% hip adduction-abduction, using Typical-split method. While the range of the relRMSE was 28%–32% for subtalar moment, 8%–9% for ankle moment, 16%–23% for knee moment, 11%–13% for hip flexion–extension, 18%–20% hip adduction-abduction when using Leave-subjects-out methods as shown in Table 3.

### 3.4 Joint moment predictions

Among all outcomes, the subtalar inversion-eversion moment was with the lowest RMSE with an average of 0.033 Nm/kg using Typical-split method and 0.081 Nm/kg when using Leave-subjects-out method. While all other joint moments showed a comparable result with an average RMSE of 0.069 Nm/kg using Typical-split method and ranging between 0.134 Nm/kg and 0.208 Nm/kg when using Leave-subjects-out method.

## 4 Discussion

This study introduced newly proposed deep neural network architectures to estimate various lower limb joint moments from inertial wearable sensors. The XCM deep neural network demonstrated highly accurate prediction using four Inertial measurement units only. This encouraging finding can therefore enable clinicians and practitioners to bypass the use of a computationally expensive Inverse dynamic approach to estimate joint moments and reduce the laborious nature of executing this process. Accordingly, our work represents a significant progress towards the use of machine learning for clinical applications.

The XCM performance was noticeably superior to all of the tested deep neural networks when predicting hip, knee, ankle, and subtalar moments with a very low level of error of an average RMSE of 0.046 ± 0.013 Nm/kg. In support of this, Camargo et al. (2022) also reported very good prediction accuracy for hip, knee, and ankle joint moments using wearable sensor data with MAE of 0.06 ± 0.02 Nm/kg. However, their predictions were based on data combined from IMU, EMG, and GON sensors, while our study proposed a model that can accurately predict joint moments using a reduced number of wearable sensors, in this case, IMUs only. The excellent prediction of the XCM could be because it extracts features related to the observed variable (using 2D convolutional filters) and time (using 1D convolutional filters) directly from the input data instead of using 2D and 1D filters in sequence, an approach used by the other tested models used in this study and their study. Utilizing a sequence of 2D and 1D convolution filters implies that temporal aspects (extracted as feature maps from 1D convolution filters) are separated from the processed features associated with observed variables (feature maps from 2D convolution filters). Consequently, the temporal features are unable to fully integrate the timing details from the input data, resulting in only partial representation of the essential information required to distinguish between distinct classes. In contrast to the 2D/1D sequential approach, XCM directly extracts features pertaining to observed variables (via 2D convolution filters) and temporal information (via 1D convolution filters) from the input data. This inclusive methodology incorporates all pertinent information, resulting in the generation of more discerning features. Consequently, XCM demonstrates improved classification performance on average, outperforming the 2D/1D sequential approach (Fauvel et al., 2021). Furthermore, Camargo et al. predicted the anticipated joint moments in future time using a fixed-size window of 250 m rather than along a full gait cycle. In the future, it would be interesting to see how well XCM can perform to predict the anticipated joint moments rather than the instant time. While comparable performance was found between the rest architectures with no noticeable differences across all joint moments with an average RMSE±SD of 0.064 ± 0.003, XCM improved joint moment prediction by 28% compared to Camargo et al. best prediction and by 23% in comparison to the rest architectures used in this study. Accordingly, our findings showcase the strength of XCM to predict joint moments during different locomotion modes using IMUs sensors only. This is particularly applicable in real-time applications that make use of joint moments in clinical assessments.

In general, the prediction of all of the tested models was comparable to previous studies (Camargo et al., 2022; B. X. W; Liew et al., 2021; Xiong et al., 2019) despite the differences in machine learning approaches, predictor types, and locomotion modes. Apart from subtalar inversion-eversion moment, the same order of magnitude for the error predicted for hip, knee, and ankle joints, was observed across all our tested models with an average RMSE±SD of 0.068 ± 0.003 Nm/kg when using the Typical-Split method and 0.165 ± 0.033 Nm/kg using Leave-subjects-out method. A similar finding was reported by Camargo et al. (2022) and Xiong et al. (2019). Camargo et al. also found no noticeable difference between the error predicted for the three joints with an average MAE±SD of 0.07 ± 0.01 Nm/kg using a shallow neural network (2 hidden layers) and 0.06 ± 0.02 Nm/kg using XGBoost algorithm during same locomotion modes used in our study. However, based on the present findings, a Typical-Split method resulted in 59% lesser RMSE than a Leave-subjects-out method, suggesting that the prediction performance of could be more optimistic. Similar to Camargo et al. study and the current study, Xiong et al. (2019) also found comparable differences in the prediction errors for hip, knee, and ankle joints during walking with an average normalized RMSE of 6.915% ± 0.657 using a shallow neural network (3 hidden layers). Unfortunately, neither nor predicted the subtalar inversion-eversion moment. However, in agreement with our findings, this particular joint moment was previously reported with a relatively lower predicted error to compare to other joints during running (Liew et al., 2021).

A recent systematic review recommended considering the variation among individuals when training a machine learning model (Xiang et al., 2022). This was confirmed by the current study. Leave-subjects-out methods increased the maximum relRMSE across all the tested models by 16% for subtalar joint moment, 4% for ankle joint moment, 16% for knee moment, 6% for hip flexion-extension moment, and 11% hip adduction-abduction moment, compared to Typical-split method. That might be because the model can find an association between the unique movement features of a subject, hence reducing prediction error. The effect can be seen more when looking at the prediction for each joint. For example, the increase in the subtalar error was the highest as the kinematics of this joint varies considerably among individuals (Brockett and Chapman, 2016) compared to other joints. Similar findings were observed in previous studies for classifications (e.g., healthy and diseased subjects) based on voice measurements (Neto et al., 2019) and activity recognition (Saeb et al., 2017), where model classification ability was reduced when ignoring subjects variability during model training. However, the purpose of the model is the most important determinant in the decision of which data should be used to train the model. For example, for subject-specific machine learning models, split based on trials or repetitions are more appropriate (Camargo et al., 2022). While if the model’s purpose is to be generalized for the public, then subject variations must be considered.

This study includes several limitations. Hyperparameter tuning has not been explored in the current study, hence, our findings can provide a more conservative estimation of the predictive performance of the machine learning models. Additionally, the temporal dependencies in our data have not been explored. The data of the current study includes multiple consecutive repetitions of multiple locomotion modes of each participant, which means data from consecutive repetitions could be highly correlated (Dorschky et al., 2023) and so the model may be overfitting to one fold (Domingos, 2012). However, Johnson et al. reported that cross-validation over five k-folds *versus* one-fold of multiple repetitions, but of one locomotion mode (sidestepping), showed a very similar average correlation when predicting knee joint moment (Johnson et al., 2019a). Future studies may further investigate the effect of cross-validation on model prediction when multiple locomotion modes are considered. The characteristics of the participants (e.g., sex, age group, health status, type of activities engaged in) may be important determinants in model prediction accuracy. A machine-learned model used for prediction purposes must be trained on data that has similar characteristics to the data needed to be predicted. Our data included all healthy participants within the same group, but various locomotion activities were included. Yet, all the activities we considered were at the same level of intensity with no vigorous activities. In term of model prediction, a recent study reported that including data on walking and running was no better than including walking or running alone (B. X. Liew et al., 2023). Nevertheless, a more diverse dataset in machine learning can benefit the model by improving generalization, reducing bias, and enhancing robustness to variations in individual characteristics. It allows the model to learn from a wider range of ages, health statuses, and activities, resulting in improved performance and fairness. However, careful data curation and evaluation are essential to address challenges and biases that may arise from the diverse dataset. Inclusion of subject characteristics into a multi-input model should be investigated in the future. Signals measured by IMU sensors are sensitive to participant anthropometrics and soft tissue characteristics (Godfrey et al., 2008), thus proper sensor placement is crucial to ensure accurate and reliable data collection. Furthermore, maintaining consistent sensor placement across multiple sessions can be challenging. Nevertheless, this challenge can be overcome by implementing standardized placement guidelines and providing training on sensor placement and calibration. Future work on assessing the effects of inter-participant variabilities on input signals for the machine learning models as well as variable selection to identify the most parsimonious combination of sensors is needed. Finally, testing and validating the use of IMU sensors combined with machine learning in uncontrolled, real-world environments require addressing real-world variability, identifying suitable ground truth measurements, performing comparative analysis, and analyzing prediction errors and uncertainties. These steps contribute to building confidence in the model’s predictive capabilities and its suitability for real-world applications.

In conclusion, XCM deep neural network can accurately predict the waveform of lower limb joint moments during walking, ramp, and stairs using inertial wearable sensors only such as IMUs. The portability of the IMU sensors is a vast advantage allowing for wider adoption in the practical setting. One of the most significant benefits of making deep neural network architectures available to field practitioners relates to the ease of estimating lower limb joint moments during different locomotion modes. Not only does this enable fast measurement, but it also facilities excellent accuracy and detailed motion analysis for athletes or other patients and so sports scientists and physiotherapists can gain insights into biomechanics, technique, and form. This information can be used to identify areas where clients may be at risk of injury, as well as to develop personalized training programs that can help them optimize their performance.

## Data Availability

The datasets generated and analyzed for this study can be found on Github: https://github.com/ZainabAltai/BiomechnicalDataSet_forMachineLearning
