# Supplementary material for: Performance of multiple neural networks in predicting lower limb joint moments using wearable sensors
Source: Front Bioeng Biotechnol. 2023 Jul 31;11:1215770. doi: 10.3389/fbioe.2023.1215770 (PMC10424442; doi:10.3389/fbioe.2023.1215770)
Supplement: Supplementary file 1 [file Table1.DOCX]

Supplementary Material

Performance of Multiple Neural Networks in Predicting Lower Limb Joint Moments using Wearable Sensors

Zainab Altai^*^, Issam Boukhennoufa, Xiaojun Zhai, Andrew Phillips, Jason Moran, Bernard X.W. Liew

*** Correspondence:** Zainab Altai: [za21920@essex.ac.uk](mailto:za21920@essex.ac.uk)

# Data used for machine learning

Data used for machine learning includes accelerations (Figure 1) and gyroscopes (Figure 2) measured by four Inertial Measurement Units (IMUs) located at the foot, shank, thigh, and trunk, and joint moments (Figure 3) of the hip, knee, ankle, and subtalar joints calculated by musculoskeletal models.

| 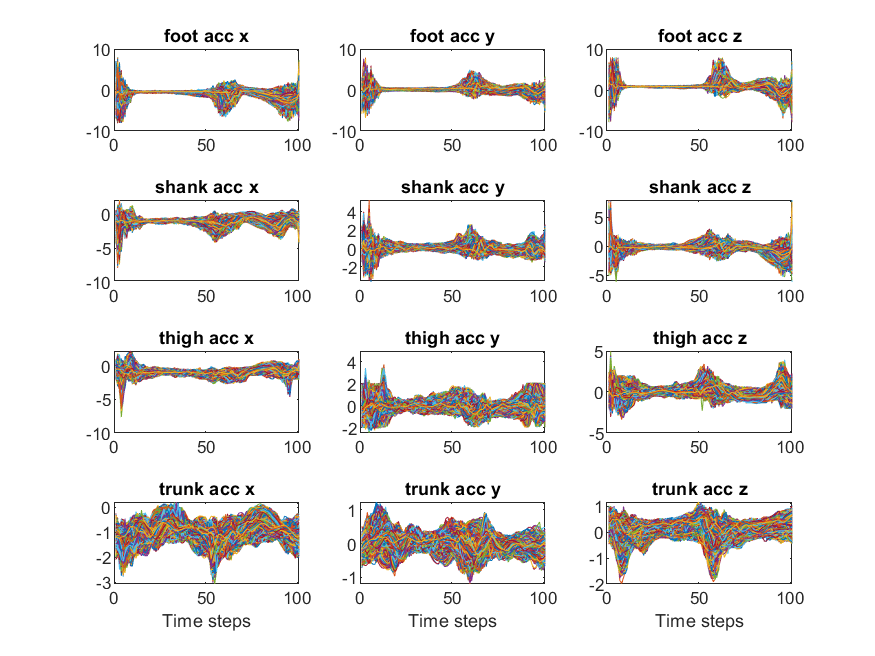 |
| --- |
| **Supplementary Figure 1.** Accelerations (acc) of the foot, shank, thigh, and trunk measured by the IMUs |

| 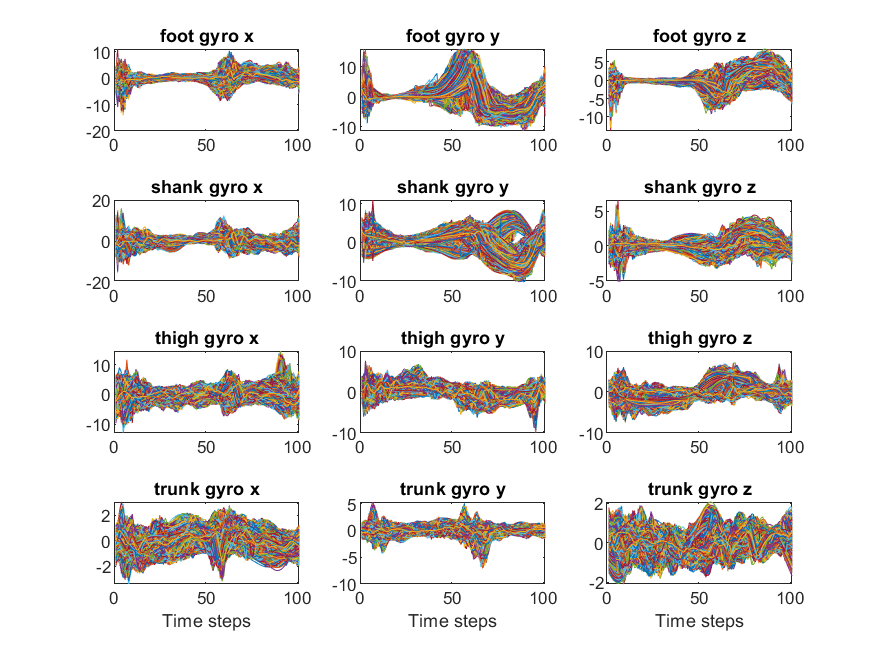 |
| --- |
| **Supplementary Figure 2.** Gyroscopes (gyro) of the foot, shank, thigh, and trunk measured by the IMUs |

| 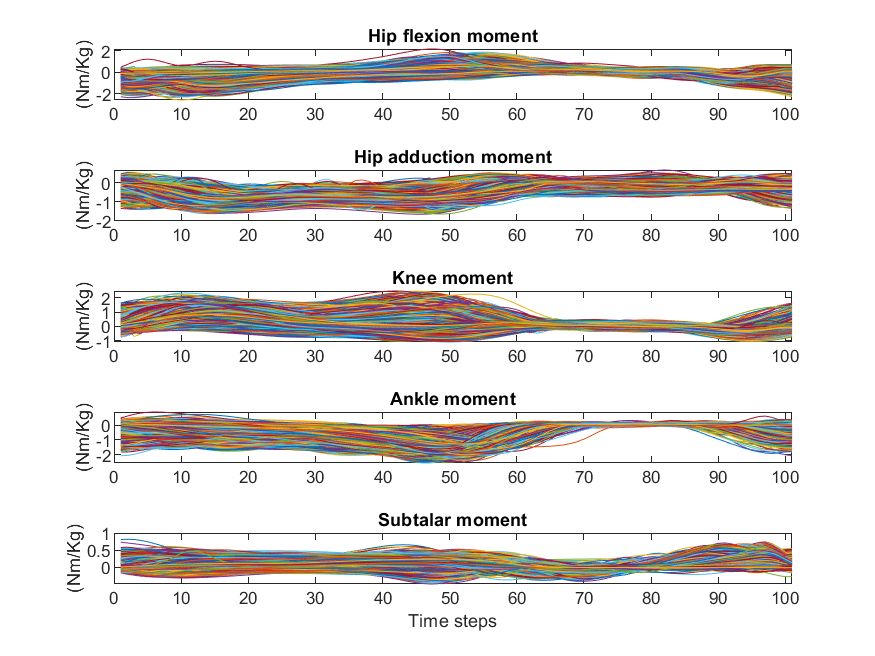 |
| --- |
| **Supplementary Figure 3.** Hip, knee, ankle, and subtalar joint moments calculated by the musculoskeletal models. |

# Performance of the machine learning models

The predicted standard deviation (SD) waveform for each of the five predicted joint moments using Typical-split (Figure 4) and Leave-subjects-out methods (Figure 5).

| 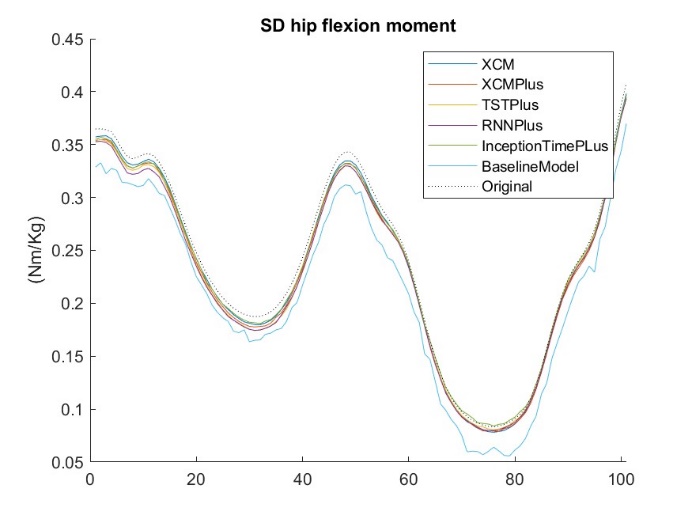 | 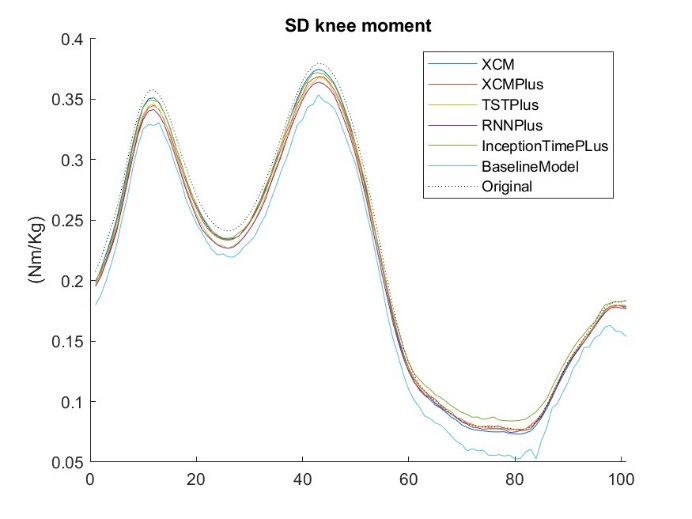 |
| --- | --- |
| 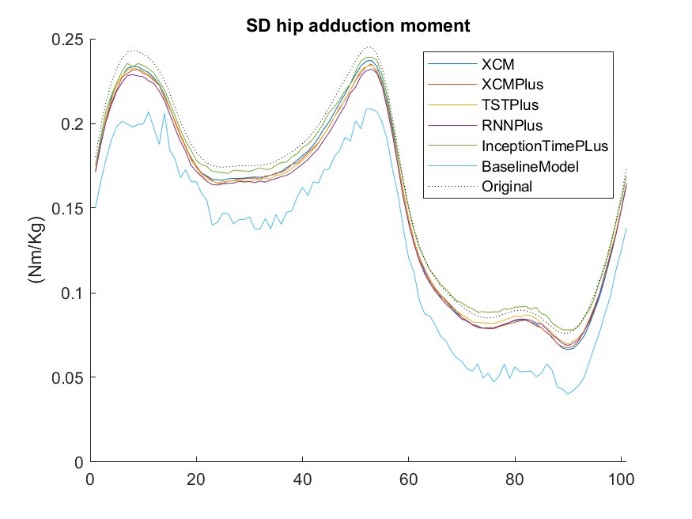 | 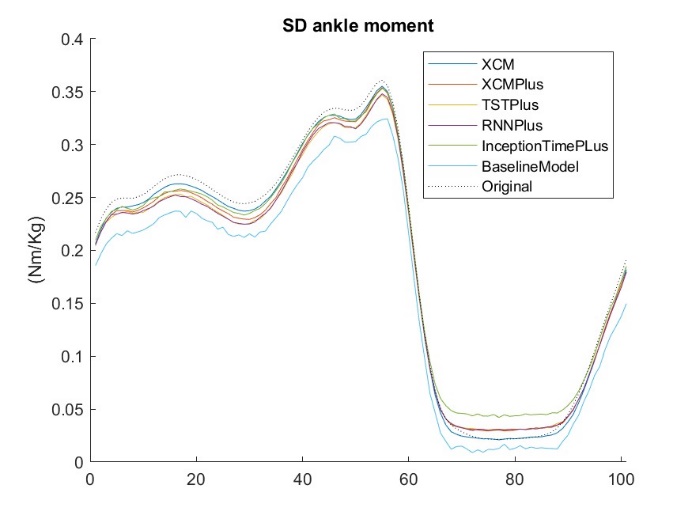 |
| 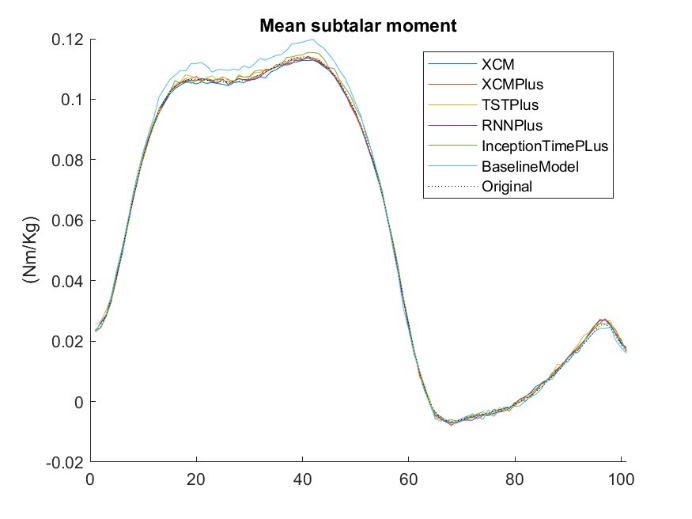 |  |
| **Supplementary Figure 4.** Standard deviation (SD) of the joint moment waveforms for the hip, knee, ankle, and subtalar joints predicted by the six machine-learning methods using Typical-split method. | |
| 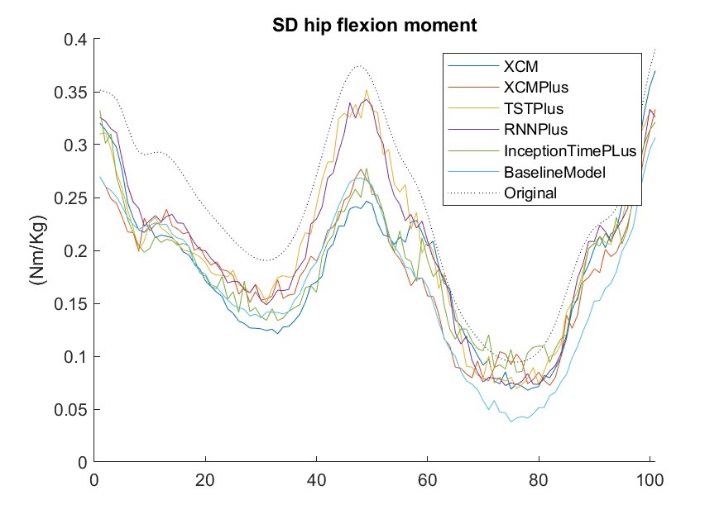 | 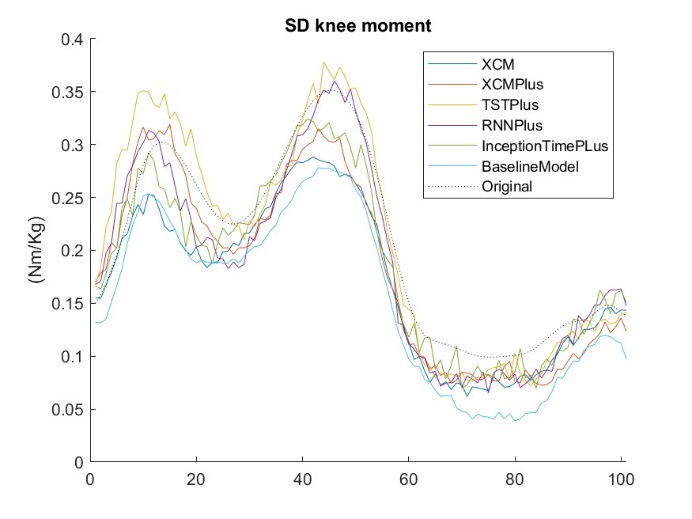 |
| 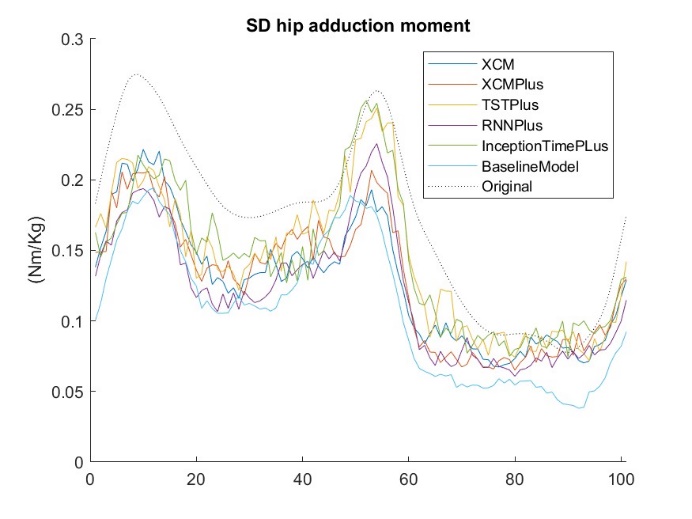 | 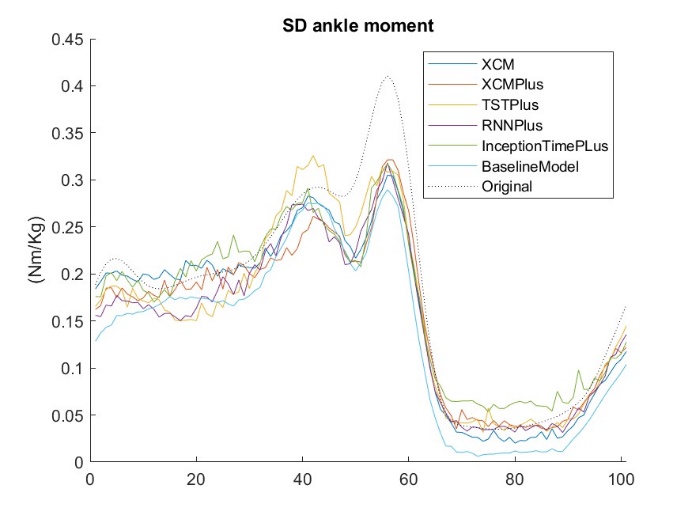 |
| 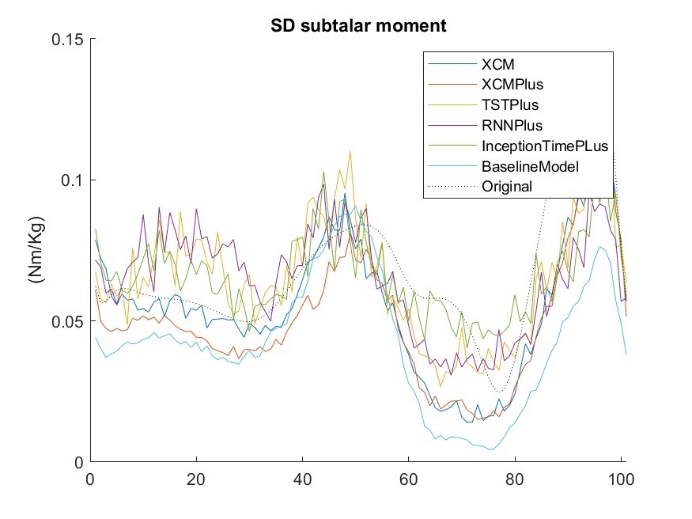 |  |
| **Supplementary Figure 5.** Standard deviation (SD) of the joint moment waveforms for the hip, knee, ankle, and subtalar joints predicted by the six machine-learning methods using Leave-subjects-out method. | |

| 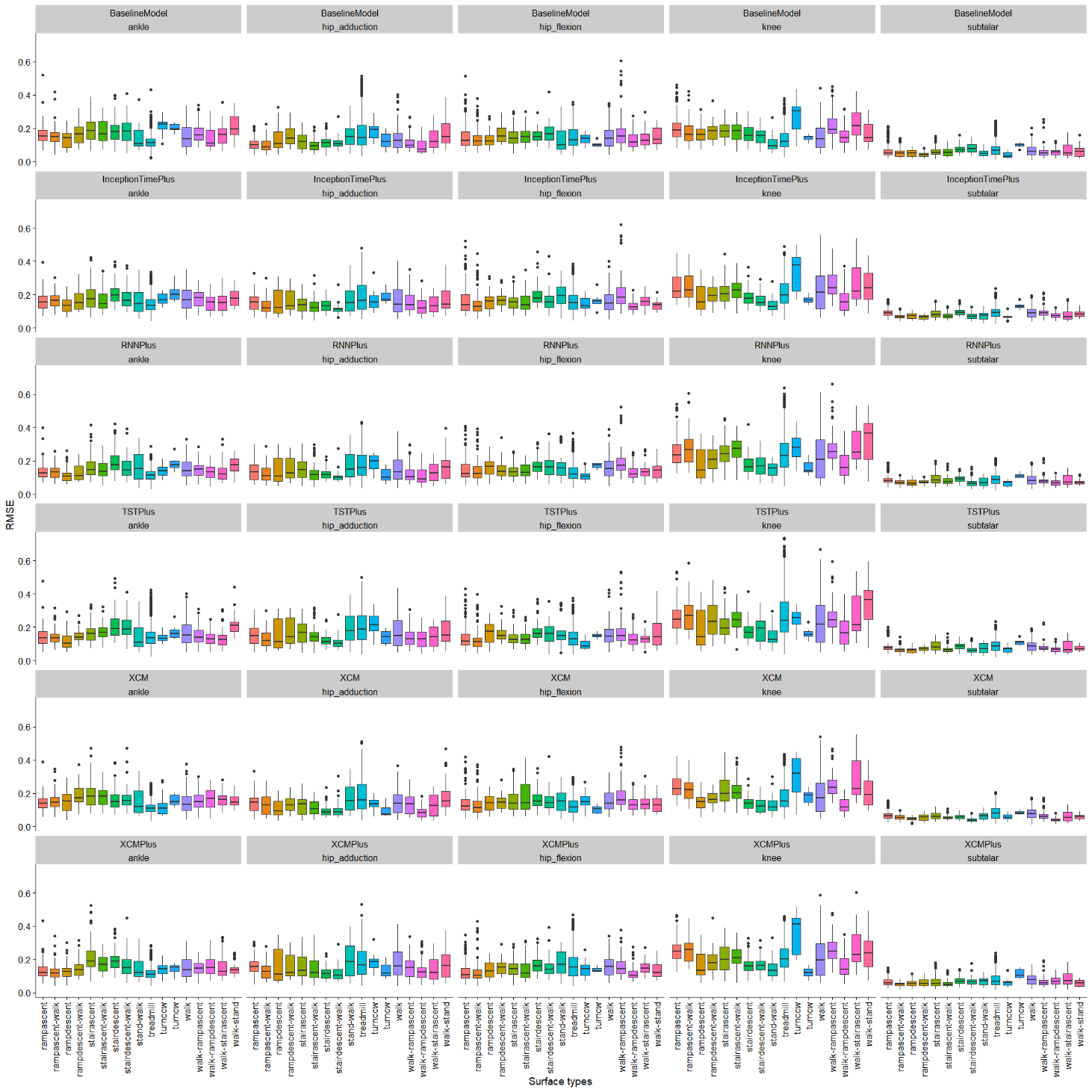 |
| --- |
| **Supplementary Figure 6.** Root mean square error (RMSE) of the hip, knee, ankle, and subtalar joint moments for different locomotion modes (Surface type) predicted by the six machine-learning methods using Leave-subjects-out method.   \| 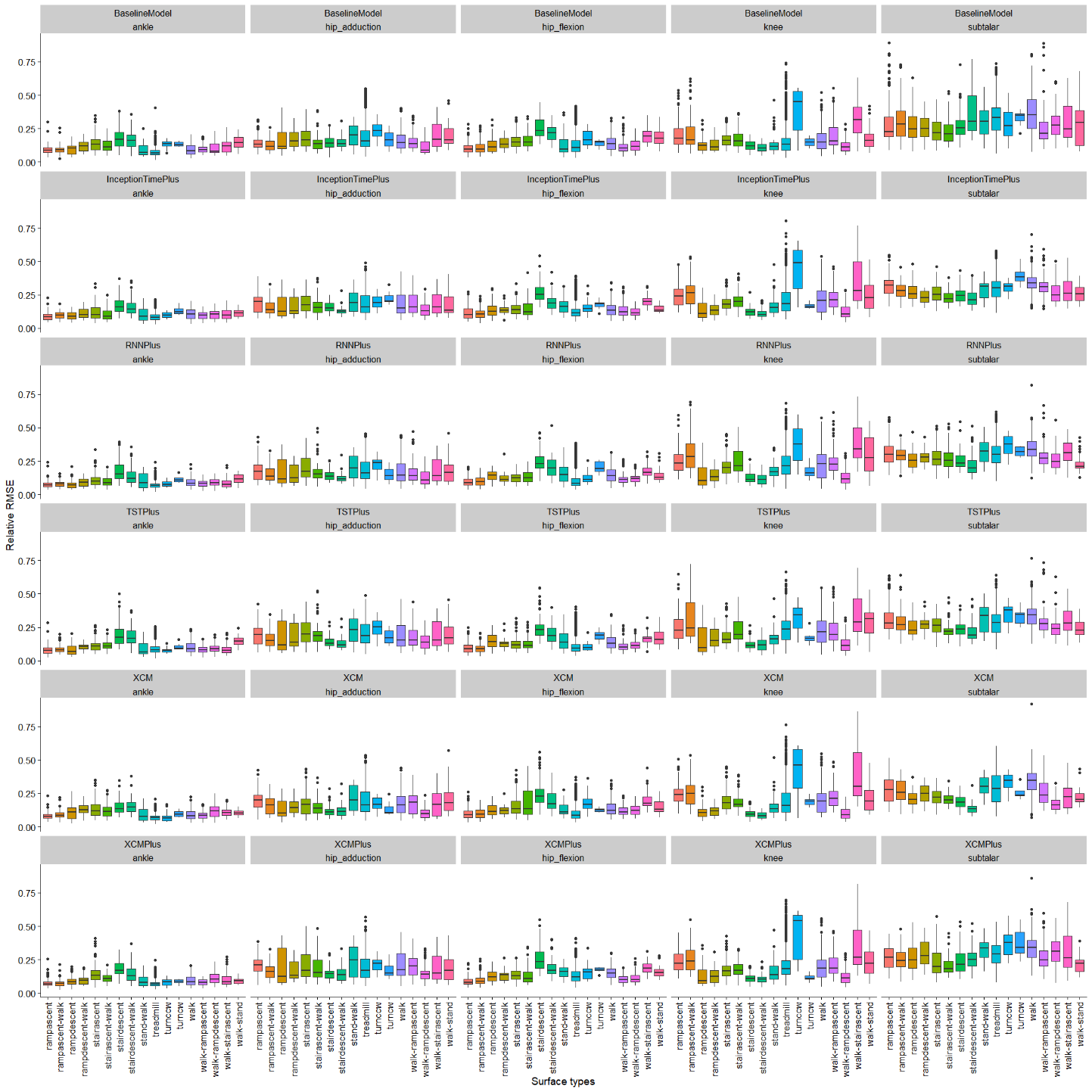 \| \| --- \| \| **Supplementary Figure 7.** Relative root mean square error (relRMSE) of the hip, knee, ankle, and subtalar joint moments for different locomotion modes (Surface type) predicted by the six machine-learning methods using Leave-subjects-out method. \| |

| 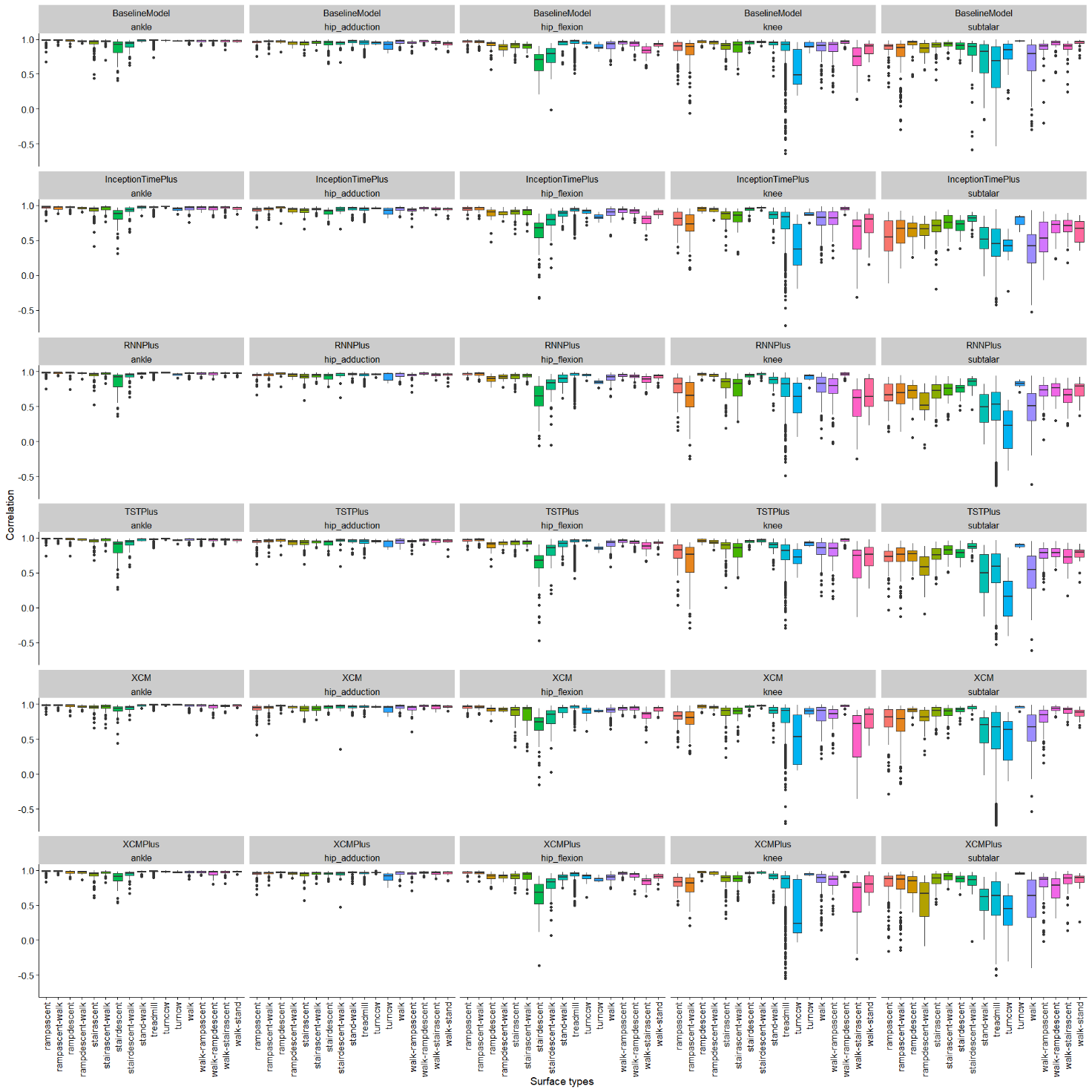 |
| --- |
| **Supplementary Figure 8.** Pearson correlation coefficient (cor) of the hip, knee, ankle, and subtalar joint moments for different locomotion modes (Surface type) predicted by the six machine-learning methods using Leave-subjects-out method. |
